# Sustainable Microbial and Heavy Metal Reduction in Water Purification Systems Based on PVA/IC Nanofiber Membrane Doped with PANI/GO

**DOI:** 10.3390/polym14081558

**Published:** 2022-04-11

**Authors:** Noha A. Elessawy, Marwa H. Gouda, Mohamed Elnouby, Safaa M. Ali, M. Salerno, M. Elsayed Youssef

**Affiliations:** 1 Computer Based Engineering Applications Department, Informatics Research Institute IRI, City of Scientific Research & Technological Applications (SRTA-City), Alexandria 21934, Egypt; elsayed168@gmail.com; 2 Polymer Materials Research Department, Advanced Technology and New Materials Research Institute, City of Scientific Research and Technological Applications (SRTA-City), New Borg El-Arab City, Alexandria 21934, Egypt; marwagouda777@yahoo.com; 3 Nanomaterials and Composites Research Department, Advanced Technology and NewMaterials Research Institute, City of Scientific Research and Technological; Applications (SRTA-City), New Borg El-Arab City, Alexandria 21934, Egypt; m_nano2050@yahoo.com; 4 Nucleic Acid Research Department, Genetic Engineering and Biotechnology Research Institute (GEBRI), City of Scientific Research and Technological Applications (SRTA-City), New Borg El-Arab City, Alexandria 21934, Egypt; Safaa.mohamedali@yahoo.com; 5 Institute for Materials Science and Max Bergmann Center of Biomaterials, TU Dresden, 01069 Dresden, Germany

**Keywords:** functionalized composite, electrospun, nanofibers, metal ions removal, adsorption, water purification

## Abstract

Effective and efficient removal of both heavy metal pollutants and bacterial contamination from fresh water is an open issue, especially in developing countries. In this work, a novel eco-friendly functional composite for water treatment application was investigated. The composite consisted of electrospun nanofiber membrane from blended polyvinyl alcohol (PVA)/iota carrageenan (IC) polymers doped with equal concentrations of graphene oxide (GO) nanoparticles and polyaniline (PANI). The effectiveness of this composite as a water purification fixed-bed filter was optimized in a batch system for the removal of cadmium (Cd^+2^) and lead (Pb^+2^) ions, and additionally characterized for its antimicrobial and antifungal properties and cytotoxicity effect. The fiber nanocomposite exhibited efficient antibacterial activity, with maximum adsorption capacity of about 459 mg g^−1^ after 120 min for Cd^+2^ and of about 486 mg g^−1^ after 90 min for Pb^+2^. The optimized conditions for removal of both metals were assessed by using a response surface methodology model. The resulting scores at 25 °C were 91.4% (Cd^+2^) removal at 117 min contact time for 89.5 mg L^−1^ of initial concentration and 29.6 cm^2^ membrane area, and 97.19% (Pb^+2^) removal at contact time 105 min for 83.2 mg L^−1^ of initial concentration and 30.9 cm^2^ nanofiber composite membrane. Adsorption kinetics and isotherm followed a pseudo-second-order model and Langmuir and Freundlich isotherm model, respectively. The prepared membrane appears to be promising for possible use in domestic water purification systems.

## 1. Introduction

Nowadays, due to fast developing industrialization, water sources on the Earth have become contaminated by different heavy metals more than their acceptable limits, which affects the ecological balance and man health. Heavy metal ions such as Cd^+2^ and Pb^+2^ are present in the soil, air, and water, and have attracted the researchers concern because of their toxicity on humans. Cd^+2^ causes kidney failure, bones diseases, and lung damage [1] whereas Pb^+2^ causes blood diseases [2]. Additionally, these elements are not biodegradable [3] and thus, easily accumulate in human beings. The accepted limit of these heavy metals was set for drinking water by the World Health Organization to 0.003 mg L^−1^ for Cd^+2^ and 0.01 mg L^−1^ for Pb^+2^. To remove heavy metals from water, several different treatment methods have been applied, such as membrane filtration [4], adsorption [5,6,7], coagulation [8], and biosorption [9]. However, adsorption is the most efficient method since it is both technically simple and inexpensive.

Among the biopolymers, iota carrageenan (IC) is an ideal candidate for drug delivery, wound healing, tissue engineering applications, and water treatment [10,11]. IC has been used as efficient green adsorbent in adsorption processes against heavy metals [12] because of its sulfonic groups that bond with positive charges of heavy metals ions by electrostatic attraction. However, the fiber structure of IC has weak mechanical properties. Therefore, a treatment of crosslinking with other synthetic polymer such as polyvinyl alcohol (PVA) is needed to enhance its performance [13,14,15,16,17].

PVA is a water-soluble green synthetic polymer with biodegradability, biocompatibility, chemical resistance, moisture absorbency, and formability of fibers [18,19], which is used in a wide range of different applications such as fuel cells [20,21], biomedical applications [22], and water treatment [19,23]. The mechanism for the PVA-IC interaction is based on water solubility of both polymers, which allows one to create strong hydrogen bonds that increase the stability of the resulting compound [18].

PANI is an electrically conducting polymer that has excellent physicochemical and environmental stability [24], in addition to remarkable advantages such as facile synthesis process, low monomer cost, and a tunable structure. PANI particles can be prepared through different chemical methods [25]. PANI is a very promising adsorbent for heavy metals due to its large amine and imine groups that can form strong interactions with them, as well as with organic pollutants. Indeed, heavy metals such as Hg^+2^, Cr^+6^, and Cd^+2^ have been removed using a variety of PANI composites with excellent adsorption capability [26].

Finally, GO is one material that, recently, has been widely used as a filler in composites, after the burst of interest in graphene during the past decade. GO, in particular, which results from addition to graphene of various oxygen-containing groups such as carboxyl, carbonyl, hydroxyl and epoxide, has demonstrated its antimicrobial activity against a number of human pathogens such as *E. coli* and *S. aureus* [27,28]

The research presented herein aimed to finding safe and effective water purification filter materials active against both heavy metals and bacteria and fungi, overcoming the continued problems of bacteria developing resistance to existing antimicrobial agents and lack of good anti-fungal agents. Therefore, in this study, we focused on the preparation and testing of PVA/IC/PANI/GO novel nanofiber composite membranes to decrease the growth of microbes and their half-life and stability at room temperature (RT), as well as to remove toxic metals such as Cd^+2^ and Pb^+2^ from contaminated water. The adsorption kinetics, isotherms, thermodynamics, and removal capacity of PVA/IC/PANI/GO nanofiber composite membrane were investigated through a series of adsorption experiments.

## 2. Materials and Methods

### 2.1. Materials

Plastic bottle wastes were taken as sources of polyethylene terephthalate (PET) and used to prepare GO. Aniline monomer, ammonium persulfate (APS), hydrochloric acid (HCl), acetone, benzimidazole 98%, PVA (MW 1,300,000), and IC were purchased from Sigma-Aldrich Co. Glutaraldehyde (GA) (Alfa Aesar, Haverhill, MA, USA, 50 wt.% in H_2_O) and 4-sulfophthalic acid (SPA) (Sigma-Aldrich, St. Louis, MO, USA, 99.9 wt.% in H_2_O) were used as covalent and ionic cross-linkers, respectively, and nonwoven polyester fabric was purchased from R.P. Industries, Gujarat, India.

### 2.2. Fabrication of the PVA/IC /PANI/GO Nanofiber Composite Membrane

The GO was obtained from PET bottle waste as mentioned in a previous study [5,6,29]. The resultant black powder was collected and crushed using a ball miller (Retsch, Germany). For the polymerization in liquid state, aniline (0.2 M) in 100 mL of 1 M HCl was oxidized with ammonium peroxydisulfate (0.25 M) in 0.1 M HCl. Solutions of a monomer and an oxidant were mixed after pre-cooling at 0 °C to start the oxidation. The appearance of green color indicated that the polymerization was completed. The solution was washed several times with acetone to remove unreacted monomer, and then dried to get the required PANI powder

For the membrane fabrication, at the beginning, 10 g of PVA (10 wt.%, 99% hydrolysis) was dissolved in H_2_O at 90 °C, and 0.05 g IC (0.5 wt.%) was dissolved in H_2_O at 80 °C. Then, a blend of the different compounds was prepared, in the PVA/IC/PANI/GO ratio of 85:5:5:5 (wt.%). After that, the polymers blend was crosslinked by using SPA (10 mL, 99.9 wt.%) as ionic crosslinker and sulfonating agent for PVA, to convert to sulfonated polyvinyl alcohol (SPVA). Then, the polymer composite was electrospun at 20 kV, with a distance between syringe tip and stationary collector of 15 cm. The nanofiber membrane was, then, crosslinked by solution method, using a 2 wt.% GA solution in 100 g of acetone for soaking during a few minutes, before finally drying at RT.

### 2.3. Characterization of Nanofiber Composite Membrane

Fourier-transformed infrared (FTIR) analysis was conducted using a spectrometer ALFA FTIR (Bruker, Billerica, MA, USA) with a range of 400–4000 cm^−1^. The Brunauer–Emmett–Teller (BET) surface area and total pore volume were measured using Barret–Joyner–Halenda (BJH) adsorption methods. A scanning electron microscope (SEM) JSM 6360LA (JEOL, Tokyo, Japan) was also used to study the morphological structure.

To investigate the stability of the membrane, pieces with size of 6 mm were immersed in 100 mL of deionized water at 35 °C for 24 h. The swelling coefficient S was calculated as follows:

(1) $$S\left(\%\right)=\frac{W_{w}-W_{d}}{W_{d}}\times 10$$

where W_w_ and W_d_ were the masses of the swollen and dry PVA/IC /PANI/GO nanofiber composite membrane, respectively.

A contact-angle analyzer (Rame-Hart Instrument Co., Succasunna, NJ, USA, model 500-FI) was employed to determine the nanofiber composite membrane’s hydrophilicity by measuring the contact angle between a water drop and the membrane surface. 

To determine the point of zero charge (pH_pzc_), 10 mg of nanofiber composite membrane was sonicated for 10 min in 10 mL solutions with pH values ranging from 3 to 9, then, shaken gently for 24 h. The final pH values of each solution were recorded, and the pH_PZC_ values were calculated.

### 2.4. Evaluation of Antimicrobial Properties of Nanofiber Composite Membrane

The prepared nanofiber composite membrane was evaluated for antimicrobial activity against different microorganisms according to two different methods. First, a susceptibility test was used based on the inhibition of microorganism diffusion on agar medium placed in Petri dishes. Bacteria at a known concentration (100 µL of bacterial culture with OD_600_ (1)) were stretched across the medium. A membrane disk was placed on the agar and was incubated for 24 h. The inhibition zone surrounding the disk was calculated. Data from this experiment was typically presented as a percentage of inhibition to indicate the levels of activity.

Additionally, to the above test on a solid surface, the antimicrobial effect of the membrane was tested in liquid solution. *E. coli* (Gram-negative bacteria), *Bacillus subtilis* (Gram-positive bacteria), *Candida albicans* (yeast), and *Aspergillus fumigatus* (fungus) were used as antimicrobial models. All microorganisms were inoculated into the LB liquid medium under 220 rpm and 37 °C, until the optical density (OD) value at 600 nm wavelength was 0.6. The bacterial solution was diluted at a volume ratio of 1:1000. A sterilized 0.01 gm of PVA/IC/PANI/GO membrane was applied to 3 mL of bacterial solution, and then incubated for 24 h at 220 rpm, 37 °C. The OD value at 600 nm was measured. The inhibition coefficient was calculated according to:

(2) $$I\left(\%\right)=\frac{\mathrm{no}.\;\mathrm{of}\;\mathrm{spore}\;\mathrm{in}\;\mathrm{treated}\;\mathrm{with}\;\mathrm{membrane}}{\mathrm{no}\;\mathrm{of}\;\mathrm{spore}\;\mathrm{from}\;\mathrm{control}\;\left(\mathrm{untreated}\;\mathrm{with}\;\mathrm{membrane}\right)}\times 100$$

### 2.5. Batch Adsorption Tests

Adsorption tests were carried out by using a batch equilibration technique. First, 50 mL of Cd^+2^ and Pb^+2^ solutions at 25 °C was added with a specific amount of prepared nanofiber composite membrane, with the help of a thermostatic shaker running at 150 rpm. Next, 1 g L^−1^ stock solutions of Cd (NO_3_)_2_.4H_2_O or Pb (NO_3_)_2_ were diluted to different degrees by adding deionized water. The remaining metal concentrations were measured using an inductive coupled plasma atomic emission spectrometer (ICP-AES) Prodigy (Teledyne Leeman Lab, Hudson, NH, USA), and turned out to be 50, 100, 150, and 200 mg L^−1^.

The amounts of adsorbed metal were calculated according to the following formulas [7]:

(3) $$q_{t}=\frac{\left(C_{0}-C_{t}\right)}{m}\times \;V$$

(4) $$q_{e}=\frac{\left(C_{0}-C_{e}\right)}{m}\times V\;$$

where q_t_ and q_e_ (mg g^−1^) were the amounts of metal adsorbed per unit weight of adsorbent at time t and equilibrium; C_0_, C_t_, and C_e_ mg L^−1^ were the metal concentrations at initial time, time t, and the equilibrium time, respectively; V (L) was the volume of metal solution; and m (g) was the amount of adsorbent. The removal efficiency R was determined as follows:

(5) $$R\left(\%\right)=\frac{\left(C_{0}-C_{t}\right)}{C_{0}}\times 100\;$$

To optimize the Cd^+2^ and Pb^+2^ removal conditions, we tried to establish a relationship between factors and responses, according to a response surface methodology model. The selected matrix for the response surface methodology followed the Box–Behnken design [30], with 13 trials.

To evaluate the adsorption process performance, three factors were used: ×1 (time, min); ×2 (initial metal concentration, mg L^−1^); and ×3 (adsorbent dose, mg), at three levels of −1, 0, and 1 [31]. Data analysis and optimization were carried out using the statistical program Statistica (StatSoft). After proper optimization of the adsorption, the kinetic and isotherm parameters of the process were characterized.

#### Static Kinetics and Isotherms Models of Adsorption

The rate and mechanism of the adsorption process could be elucidated based on kinetic studies [7], with different initial concentrations of Cd^+2^ and Pb^+2^ varied from 50 to 200 mg L^−1^ in Tables S2–S5. The pH was fixed at 6 and the dose of adsorbent was 2 mg mL^−1^, while the desorption time was varied from 0 to 240 min. In order to elucidate the adsorption kinetics, the pseudo-first-order, pseudo-second-order, and intraparticle diffusion models were applied, as illustrated in Table 1. Additionally, to describe how the adsorbate interacts with adsorbents and to give a thorough understanding of the nature of interaction, the adsorption isotherm models were tested to validate the metal uptake behavior of the prepared PVA/IC/PANI/GO nanofiber composite membrane using Langmuir and Freundlich isotherms.

### 2.6. Reusability test

To investigate the reusability of the prepared PVA/IC/PANI/GO membrane, 10 mg of membrane was mixed with 50 mL of 20 mg L^−1^ Cd^+2^ and Pb^+2^. The mixture was shaken for 15 min. After adsorption, the membrane was separated. Then, 0.01 M HCl was added, and the solution was shaken again for 15 min. Finally, the membrane was repeatedly washed using 70% (*v*/*v*) ethanol aqueous solution, until Cd^+2^ and Pb^+2^ was no longer detected in the solution. Five cyclic adsorption–desorption processes were conducted to study the reusability.

## 3. Results and Discussion

### 3.1. Characterization of the Prepared PVA/IC/PANI/GO Nanofiber Composite Membrane

**Figure 1:**
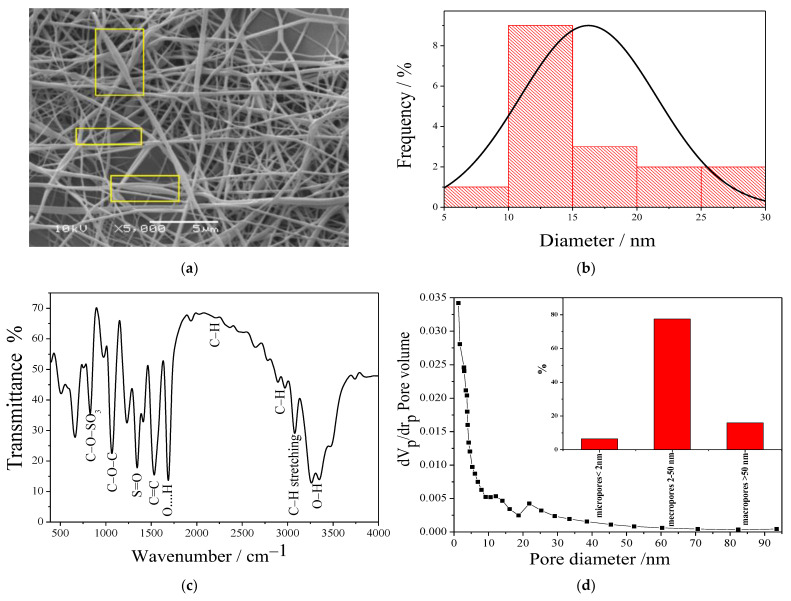
Characterization of the PVA/IC/PANI/GO nanofiber composite membrane: (**a**) Representative SEM image (rectangular frames identify spindle objects around fibers, likely to be associated with GO fillers); (**b**) frequency distribution histogram of fibers diameter; (**c**) FTIR transmittance spectrum; (**d**) average pore diameter with the inset representing the percentage of pore types in the resulting PVA/IC/PANI/GO nanofiber composite membrane.

As shown in Figure 1a, SEM imaging of the membrane demonstrated bead-free, smooth, and uniform nanofibers. Some bulges with spindle shape were observed (see regions inside rectangular frames), which may be ascribed to the GO sheets probably rolled inside along the main fiber axis, and mainly hidden by the surrounding polymer. The average diameter of nanofibers was determined by measuring the diameters of random selected fibers via ImageJ software (*n* = 17). The size distribution was roughly monomodal with a mean ± standard deviation of 19 ± 3 nm, as shown in Figure 1b.

FTIR spectroscopy was used to investigate the functional groups of the prepared PVA/IC/PANI/GO membrane, as shown in Figure 1c. The characteristic bands of IC appear around 845 which is attributed to the presence of C–O–SO_3_ of D-galactose-4-sulfate, and appear around 1260 cm^−1^ which refers to the presence of S=O [14]. The band around 1530 cm^−1^ is attributed to the C=C stretching distortion of some quinoid rings and benzenoid rings in PANI [25]. A strong band appearing at 1650 cm^−1^ can be assigned to the hydrogen bonds formed between hydroxyl groups in IC and PVA and the oxygen functional groups of the GO. A band appearing at 2200 cm^−1^ is usually assigned to the C–H bond of the polymers in the electrospun membranes [32]. The bands around 1650–2000 cm^−1^ are attributed to C–H bending in the aromatic structure of the sulfophthalic acid (SPA), which ensures the ionic crosslinking of polymers by SPA [33]. The bands between 2830–2695 cm^−1^ are attributed to the C–H of aldehyde groups, confirming the covalent crosslinking of polymers by glutaraldehyde (GA) [34]. The board bands appearing between 3078–3470 cm^−1^ is characteristic of the stretching –OH groups of the PVA and GO [35].

The BET analysis was used to investigate the specific surface area of the membrane, which was calculated to be 139 m^2^g^−1^. The pore size distribution of the prepared membrane is shown in Figure 1d, and can be described as a population of mesopores with size ranging between 2 nm and 50 nm, as shown in the inset Figure 1d. It is expected that such a porous structure can facilitate adsorption processes, for example, aiming at effectively subtracting a high amount of metal ions from the water [36].

**Figure 2:**
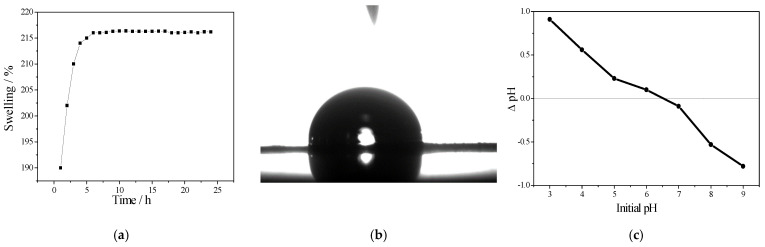
(**a**) Swelling response; (**b**) contact angle; (**c**) point of zero charge curve of the prepared PVA/IC/PANI/GO nanofiber composite membrane.

The degree of stability of the prepared PVA/IC/PANI/GO nanofiber membrane was investigated using the swelling test, as described in Section 2.3. The resulting swelling coefficient values are shown in Figure 2a. A plateau appeared in the amount of swelling after around 6 h. These results indicated that the water penetration was restricted by chemical crosslinking of PVA with IC due to intermolecular strong interaction, in addition to obstruction of the membrane pores by polyaniline as well as to its hydrophobic nature [37]. This was in contrast with the contact angle result, which was 84.4°, as shown in Figure 2b, since GO nanoparticles are more hydrophilic than PANI [6]. This suggests that incorporation of GO leads to an increase in hydrophilicity of the membrane. The point of zero charge of the prepared PVA/IC/PANI/GO nanofiber membrane is revealed in Figure 2c under various pH values. It is clear that the point of zero charge increases with increasing pH. The pH_zpc_ value of the prepared PVA/IC/PANI/GO nanofiber membrane was determined to be 6.5. Notably, the surface electrical charge of the membrane is neutral at pH equal 6.5; however, at pH less than 6.5 the membrane surface becomes positively charged and above this value it becomes negatively charged.

### 3.2. Evaluation of Antimicrobial Properties

Large zones of inhibition growth obtained by the disc diffusion method were observed against *E. coli*, *Bacillus subtilis*, *Candida albicans*, and *Aspergillus fumigatus* on the prepared membrane. This disk diffusion (the zone of inhibition) method is probably the oldest type of test existing for this purpose, and possibly, such a widely used old method is due to its ease of execution and low cost.

**Figure 3:**
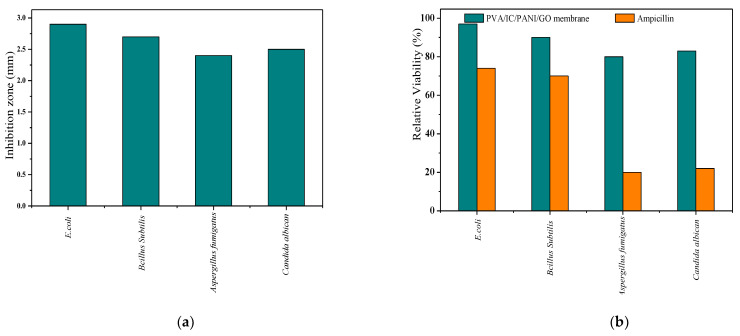
(**a**) Antimicrobial evaluation (inhibition growth diameter) obtained by using disc diffusion method; (**b**) relative viability of (*E. coli*, *Bacillus subtilis*, and *Candida albicans*) after 24 h and *Aspergillus fumigatus* after 5 days incubating with ampicillin (orange bars) as compared with that resulting from incubating on the membrane (blue bars).

According to this observation, it was concluded that the membrane has good antibacterial effect, which is ascribed to the antibacterial properties of GO [38,39]. Furthermore, for testing the antifungal activity with the same method, the membrane was also inoculated with *Aspergillus fumigatus* and *Candida albicans*. The calculated inhibition coefficients according to Equation (10) are presented in Figure 3a.

Relative viability of the different microbes after 24 h (for *E. coli*, *Bacillus subtilis*, and *Candida albicans* and 5 days for *Aspergillus fumigatus*) of incubation with the membrane and ampicillin (100 mg L^−1^) were also determined, and these results were compared with the inhibition disc results in Figure 3b. This comparison shows that the PVA/IC/PANI/GO nanofiber composite membrane has excellent antibacterial properties, on the same level as those provided by ampicillin.

Residual activity of the membrane after preservation in RT for a long time was also determined by using the plate with disc diffusion. After 70 days, no bacterial or fungal organisms could grow around the membrane, meaning that it could remain active for such a long time.

### 3.3. Surface Chemistry and Effect of Solution pH on Adsorption

**Figure 4:**
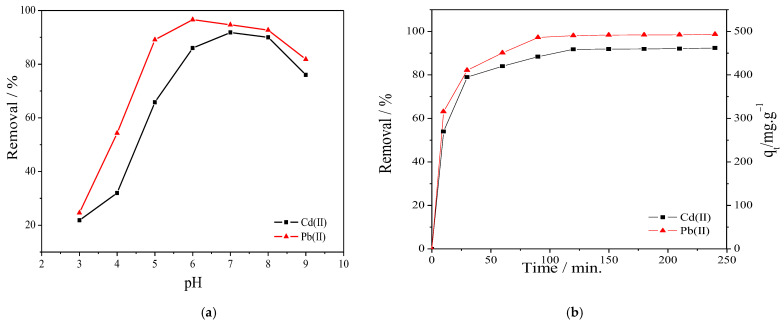
(**a**) Effect of solution pH on the removal of Cd^+2^ and Pb^+2^ (at C_0_ 100 mg L^−1^, membrane area 25 cm^2^, temperature 25 °C, time 60 min, and volume 50 mL); (**b**) effect of contact time on the removal of Cd^+2^ and Pb^+2^ (at C_0_ 100 mg L^−1^, membrane area 25 cm^2^, temperature 25 °C, and solution pH equal 6).

The prepared PVA/IC/PANI/GO nanofiber composite membrane, which is composed of cross-linked PVA with IC ionically and chemically using sulfophthalic acid (SPA) and glutaraldehyde (GA), offers many negatively charged sulfonic acid groups [14] in addition to negatively charged carboxylic, and hydroxyl groups on GO. All these functionalities make the membrane suitable for adsorption of cations, however, the pH of the solution can affect the interaction between the adsorbate and the charges on the adsorbent surface, and therefore, is one important parameter controlling the adsorption process. The influence of solution pH on adsorption of Cd^+2^ and Pb^+2^ onto prepared membrane was evaluated in the range from 3 to 9, to determine the optimal pH value. As shown in Figure 4a, the metal cation removal efficiency increased when the pH increased from 3 to 7. This effect may be ascribed to the point that at low pH values the membrane surface is surrounded by numerous hydronium ions that compete with metal ions; however, by increasing the pH, the sulfate and carboxylic groups in the polymeric chain are completely ionized [40] and the electrostatic attraction between metal ions and membrane surface is increased, resulting into gradually improved removal efficiency. However, once pH goes above 7, adsorption decreased due to formation of hydroxide forms Cd(OH)_2_ and Cd(OH)_3_^−^, as well as Pb(OH)_2_ and Pb(OH)_3_^−^ that begin to precipitate [6,41]. Additionally, one can observe from Figure 4a that at pH 6 the membrane reached the highest removal for Pb^+2^ ions, while at pH 7 it was better for Cd^+2^ removal. In the following experiments, for removal of both cations, we selected the single pH value of 6.

Next, we characterized the time dependency of absorption capacity of the membrane, see Figure 4b. Here, removal was evaluated at different times, with the shortest time being only 10 min, and the following ones after steps of 40 min to each other. Apparently, adsorption capacity increased with time up to a plateau after approximately 90 min. The steady-state situation being reached after this period may be due to a balance occurring between adsorption and desorption of metal ions.

### 3.4. Optimization of the Adsorption Process by the Box–Behnken Design Analysis

We aimed to optimizing the adsorption processes by means of a Box–Behnken design analysis. Three variables were selected, namely time, initial metal concentration, and adsorbent dose, (for details see Table S1 in Supporting Information). As mentioned above, the solution pH, in all cases, was kept constant during the whole reaction time at a value of 6, while the temperature was set to 25 °C.

To model the statistical relationship between variables and responses, namely the yield of removal Y, we assumed quadratic dependence, according to the following equations:

Y_Cd_ = 91.4 + 3.3375x_1_ − 5.225x_2_ + 8.9125x_3_ − 1.2875x_1_^2^ − 5.0625x_2_^2^ − 5.4875x_3_^2^ + 1.55x_1_x_2_ − 1.025x_1_x_3_ + 2.5x_2_x_3_ (11)

Y_Pb_ = 97.19 + 2.8375x_1_ − 3.5625x_2_ + 5.725x_3_ − 2.0575x_1_^2^ − 2.5075x_2_^2^ − 2.8325x_3_^2^ − 0.275x_1_x_2_ − 1.2x_1_x_3_ + 2.7x_2_x_3_ (12)

where X_1_, X_2_, and X_3_ are the abovementioned variables of contact time, initial metal concentration, and adsorbent dose, respectively. 

As a result of this analysis, the optimum conditions appeared to be as follows: 117 min contact time, 89.5 mg L^−1^ initial concentration, and 0.0084 mg (29.6 cm^2^) of membrane for Cd^+2^ to achieve 91.4% removal and 105 min contact time, 83.2 mg L^−1^ initial concentration and 0.0087 mg (30.9 cm^2^) of membrane for Pb^+2^, in which case 97.19% removal was achieved.

**Figure 5:**
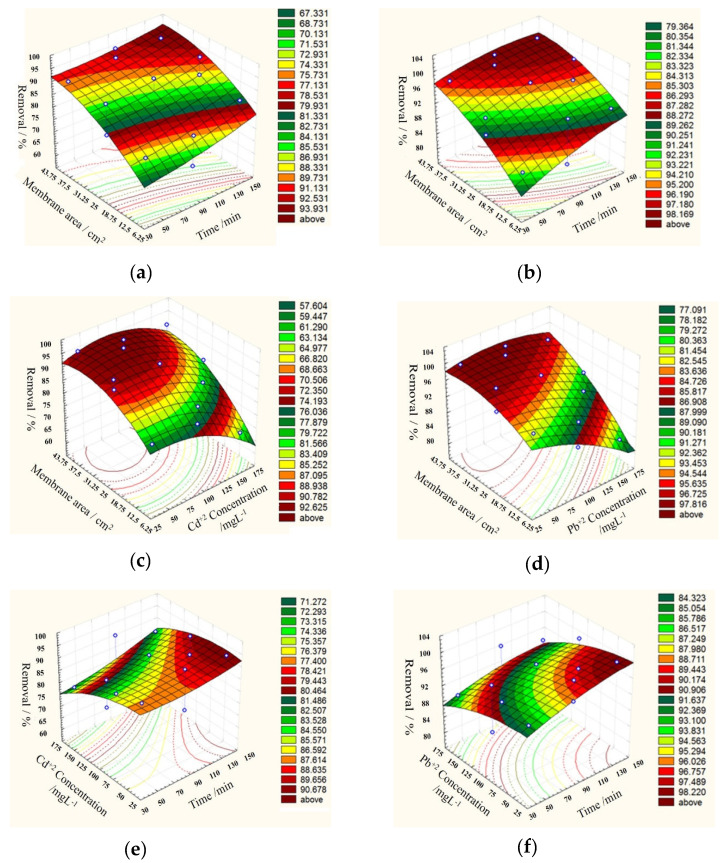
3D surface plots of Cd^+2^ (**a**,**c**,**e**) and Pb^+2^ (**b**,**d**,**f**) removal efficiency (%) on the prepared PVA/IC/PANI/GO nanofiber composite membrane.

The 3D surface plots reported in Figure 5 present the results of the Box–Behnken design analysis, and show the type of interaction between the tested variables. It appears that the removal efficiency decreased with an increase in initial metal concentrations from 50 to 150 mg L^−1^, while it increased by increasing the contact time, tending to level to an approximately constant value for Pb. This observation revealed that, in the beginning, the metal ions were adsorbed externally, and the adsorption rate increased rapidly. When the external surface was saturated, the metal ions adsorbed through the membrane pores and finally reached a constant value where no more adsorption occurred.

### 3.5. Kinetic Models of Adsorption

**Figure 6:**
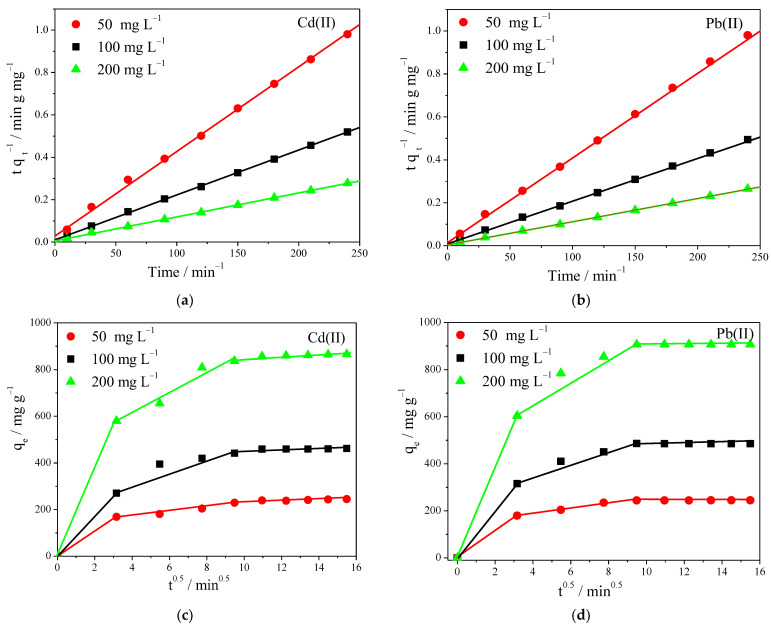
(**a**,**b**) Pseudo-second-order kinetic model; (**c**,**d**) intraparticle diffusion kinetic model for Cd^+2^ and Pb^+2^ adsorption process onto the prepared PVA/IC/PANI/GO nanofiber membrane at pH = 6. C_0_ 100 mg L^−1^, membrane area 25 cm^2^, temperature 25 °C, and volume 50 mL.

Adsorption of Cd^+2^ and Pb^+2^ on the fabricated nanofiber membranes reached equilibrium after 120 min and 90 min, respectively. We tried to gain a deeper understanding of the adsorption mechanism by using kinetic models of the process, for various initial metal concentrations of 50, 100, and 200 mg L^−1^. The kinetic data were fitted by the pseudo-first-order, pseudo-second-order equation, and intraparticle diffusion model, and the respective plots and relevant parameters are presented in Figure 6 and Table S6.

By comparing the adsorption capacities calculated using the different models with the experimentally observed ones, it was found that the pseudo-first-order model provided values far from the experimental ones, whereas the pseudo-second-order model provided values in good agreement, with correlation coefficients (R^2^) consistently higher than those of pseudo-first-order model.

The adsorption obeying closely the pseudo-second-order model, meant that the controlling rate step was chemisorption, and the rate of adsorption for both ions depended on the accessibility to adsorption sites on the surface of adsorbent surface.

In order to further clarify the diffusion mechanism of Cd^+2^ and Pb^+2^ into the membrane, an intraparticle diffusion model was used, at different initial concentrations. As shown in Figure 6c,d, the adsorption mechanism for Cd^+2^ and Pb^+2^ basically occurred in three separate steps: (i) external diffusion of the ions from the aqueous solution to the membrane surface, (ii) gradual adsorption into the membrane pores, and (iii) reaching a final equilibrium whereas an adsorption reaction occurred with the functional groups within the pores. However, the adsorption rate mainly depended on the first two steps, which most likely depended on electrostatic interactions, followed by a relatively slow step, which corresponded to the intraparticle diffusion of ions into the membrane [42].

### 3.6. Static Adsorption Isotherm Models

**Figure 7:**
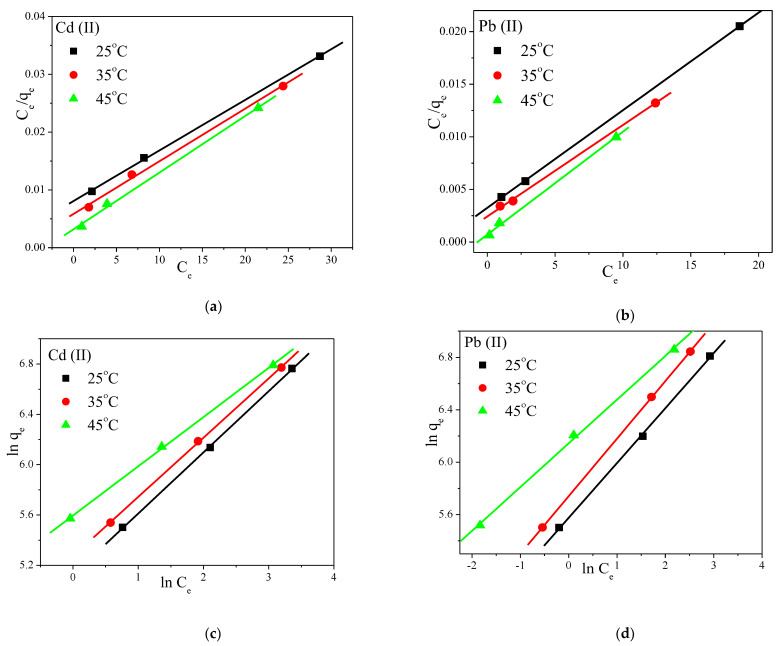
(**a**,**b**) Langmuir and (**c**,**d**) Freundlich adsorption isotherm fits for the adsorption of Cd^+2^ and Pb^+2^ into the prepared PVA/IC/PANI/GO nanofiber composite membrane at pH = 6. C_0_ 100 mg L^−1^, membrane area 25 cm^2^, volume 50 mL, for 60 min.

Langmuir and Freundlich adsorption isotherm models [41,42] were also used, at different initial concentrations and three different temperatures (25 °C, 35 °C, and 45 °C), and the obtained results are presented in Figure 7 and Table S7. The Langmuir model best represented the adsorption data, suggesting that Cd^+2^ and Pb^+2^ ions adsorption is monolayer coverage. The highest removal capacity for Cd was 1030 mg g^−1^, while for Pb it was 1078 mg g^−1^, and the adsorption was more effective at high temperatures. Furthermore, for the Freundlich model, the values of 1/n, which measure the surface heterogeneity for absorption, fluctuated between 0 and 1, pointing to a greater heterogeneity as the values approached zero. It was also concluded that heterogeneous physisorption and chemisorption processes occurred, which was in agreement with the intraparticle diffusion kinetics and with the fact that both metal ions adsorbed into the membrane mainly through inner surface with dominating intraparticle diffusion.

As shown in Table 2, various adsorbent materials were evaluated to remove Cd^+2^ and Pb^+2^ from water.

**Table 2:** A look at the effectiveness of PVA-based adsorbent materials for removing Cd^+2^ and Pb^+2^ from water.

Adsorbent Nanomaterials	Optimum Adsorption Condition (Temperature °C, pH)	Adsorbate Initial Concentration (mg L^−1^)	Removal (%)	Reference
β-Cyclodextrin(β-CD)/chitosan (CS)/polyvinyl alcohol (PVA) nanofiber membrane	30 °C	Pb^+2^, 20.72 mg L^−^^1^	100% within 10 min	[42]
**Titania PVA-alginate beads**	25 °C, pH 7	Cd^+2^, 50 mg L^−1^	100% after 3 h	[43]
**poly(β-cyclodextri) (Poly-βCD) sub-micrometric fibers**	pH 7.8	Cd^+2^, 10 mg L^−1^	6% after 24 h	[44]
KFC/CNT/PVA + Glu ternary blend	pH 5	Cd^+2^, 200 mg L^−1^	86.9% after 5 h	[45]
PVA/IC/PANI/GO nanofiber composite membrane	25 °C, pH 6	Cd^+2^, 100 mg L^−1^ Pb^+2^, 100 mg L^−1^	91.8% after 2 h 97.2% after 1.5 h	This work

### 3.7. Desorption–Adsorption Tests

**Figure 8:**
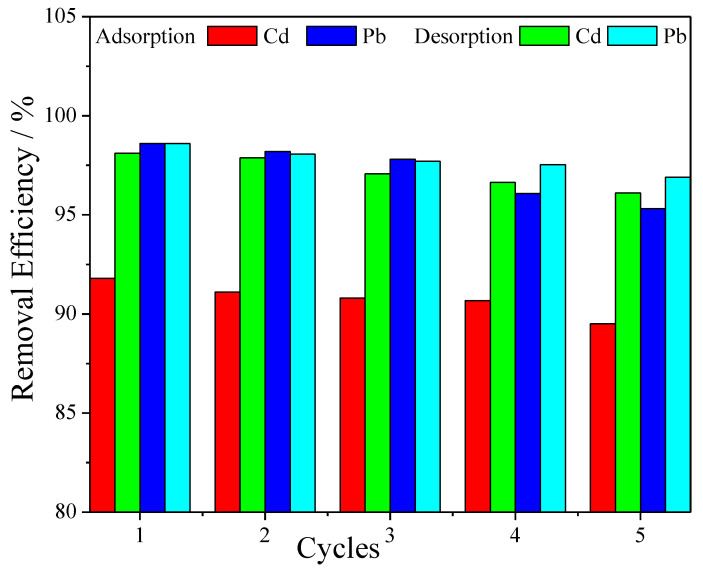
Adsorption–desorption cycles of metal ions into PVA/IC/PANI/GO nanofiber composite membrane with an initial concentration 20 mg L^−1^, membrane area 25 cm^2^, pH 6, and temperature 25 °C.

In order to evaluate the reusability and structure stability of the nanofiber composite membrane, five adsorption–desorption cycles were carried out. As shown in Figure 8, it was found that even after this repeated use the removal efficiency was still high, as only a slight decrease in the removal coefficient occurred after each desorption step, around 1%. This is a good indication for the possible use of the membrane in real-water purification systems to be industrially produced on a large scale.

## 4. Conclusions

A novel membrane material for water purification systems was fabricated, resulting from electrospinning nanofibers of PVA/IC doped with PANI/GO. After morphological and compositional characterization, the membranes were tested for removal of Cd and Pb ions, as well as for biocidal effect against bacterial and fungal strains. Against the selected fungi, the biocidal action of the membrane was even better than that of ampicillin antibiotic at standard concentration. The performance for removal of metal ions was particularly investigated, and optimization of the removal efficiency by the membrane was carried out according to a response surface methodology design. Removal coefficients above 90% were reached for both metals, after a response time of at least 90 min. The analysis of membrane absorbance after different kinetic models provided additional insight into the removal mechanism, which emerged to be due to a combination of electrostatic adsorption and chemisorption by the functional groups present inside membrane pores, due to both nanofiber composition and doping, enabling different interactions with the pollutant metal ions. According to the Langmuir isotherm model, the highest removal capacity for Cd was 1030 mg g^−1^, while for Pb it was 1078 mg g^−1^, and the adsorption was more effective at high temperatures. A demonstration of good reusability was also carried out by observing five repeated absorption/desorption cycles of the membrane, which exhibited only minor loss in performance, i.e., of the order of 1% for each cycle. This study demonstrates the good potential of the designed material for application in consumer systems for domestic water purification.

## Figures and Tables

**Table 1:** Kinetics and isotherms models of adsorption process.

Model	Linear Form	Equation No.	Plot	Parameters and Constants
Pseudo-first-order kinetic	ln (q_e_ − q_t_) = ln q_e_ − k_1_t	(6)	ln(q_e_−q_t_) vs. t	Where k_1_ is the pseudo-first-order adsorption rate constant; q_e_ is the amount of metal adsorbed at saturation per gram of adsorbent (mg g^−1^), q_t_ is the amount of metal adsorbed at time t per gram of adsorbent (mg g^−1^)
Pseudo second-order kinetic	$\frac{t}{q_{t}}=\left[\frac{1}{k_{2}qe^{2}}\right]+\frac{1}{q_{e}}\;t$	(7)	t/q_t_ vs. t	Where k_2_ is the pseudo-second-order adsorption rate constant
Intraparticle diffusion kinetic	q_t_ = k_i_ t ^½^ + C	(8)	q_t_ vs. t^1/2^	Where k_i_ (mg g^−1^ min^−1/2^) is the intraparticle diffusion rate constant, which is the slope of the straight line of q_t_ versus t^1/2^; C is the value of intercept, which is a constant reflecting the significance of the boundary layer or mass transfer effect
Langmuir isotherm	$\frac{q_{e}}{C_{e}}=\frac{1}{K_{L}q_{m}}+\frac{C_{e}}{q_{m}}$	(9)	(C_e_/q_e_) vs. C_e_	Where q_e_ is the solid-phase concentration in equilibrium with the liquid-phase, concentration C_e_ is expressed in mole L^−1^, q_m_ is the maximum monolayer adsorption capacity (mg g^−1^), and K_L_ is an equilibrium constant (L mol^−1^)
Freundlich isotherm	$\ln q_{e}=\ln K_{f}+\frac{1}{n}\;\ln C_{e}$	(10)	ln q_e_ vs. ln C_e_	Plotting ln q_e_ versus ln C_e_ gives a straight line with slope of 1/n, where n is a constant related to adsorption intensity and its magnitude shows an indication of the favorability of adsorption; the intercept is ln K_f_ where K_f_ is constant (function of energy of adsorption and temperature).

## Data Availability

All data generated or analysed during this study are included in this published article and its Supplementary Information files.

## Supplementary Materials

The following supporting information can be downloaded at: https://www.mdpi.com/article/10.3390/polym14081558/s1, Table S1: The Box–Behnken design matrix and results for the three variables that influenced the removal (%) of experimental and predicted values for Cd (II) and Pb (II) using prepared membrane at solution pH 6, Table S2: Regression statistics of Cd removal, Table S3: ANOVA of the Cd removal process, Table S4: Regression statistics of Pb removal, Table S5: ANOVA of the Pb removal process, Table S6: Parameters and determination coefficients of the kinetic models for Cd (II) and Pb (II) adsorption on prepared nanofiber membrane, Table S7: Adsorption isotherm parameters for Cd (II) and Pb (II) adsorption on prepared nanofiber membrane.
