# Supplementary material for: Sustainable Microbial and Heavy Metal Reduction in Water Purification Systems Based on PVA/IC Nanofiber Membrane Doped with PANI/GO
Source: Polymers (Basel). 2022 Apr 11;14(8):1558. doi: 10.3390/polym14081558 (PMC9025637; doi:10.3390/polym14081558)
Supplement: Supplementary file 1 [file polymers-14-01558-s001.zip › polymers-1646501-supplementary.pdf]

Supplementary Material

Sustainable microbial and heavy metal reduction in water purification systems based on PVA/IC nanofiber membrane doped with PANI/GO

Noha A. Elessawy*[1], Marwa H. Gouda[2], Mohamed Elnouby[3], Safaa M. Ali[4], M. Salerno[5,*], M. Elsayed Youssef [1]

[1] Computer Based Engineering Applications Dept., Informatics Research Institute IRI, City of Scientific Research & Technological Applications (SRTA-City), 21934 Alexandria, Egypt.
E. mail: **nony_essawy@yahoo.com , elsayed168@gmail.com**

[2]Polymer Materials Research Department, Advanced Technology and New Materials Research Institute, City of Scientific Research and Technological Applications (SRTA-City), New Borg El-Arab City, 21934 Alexandria, Egypt.
E. mail:**marwagouda777@yahoo.com**

[3] Nanomaterials and Composites Research Department, Advanced Technology and New Materials Research Institute, City of Scientific Research and Technological Applications (SRTA-City), New Borg El-Arab City, 21934 Alexandria, Egypt.
E. mail:**m_nano2050@yahoo.com**

[4]Nucleic Acid Research Department, Genetic Engineering and Biotechnology Research Institute (GEBRI), City of Scientific Research and Technological Applications (SRTA-City), New Borg El-Arab City, 21934 Alexandria, Egypt.
E. mail: **Safaa.mohamedali@yahoo.com**

[5]Institute for Materials Science and Max Bergmann Center of Biomaterials, TU Dresden, 16163 Dresden, Germany
E. mail: **marco.salerno@tu-dresden.de**

Table S1: The Box-Behnken design matrix and results for the three variables that influenced the removal (%) of experimental and predicted values for Cd (II) and Pb (II), using the prepared membrane at solution pH 6.

Trial	Time (X1; min)	Adsorbate initial concentration (X2; mg/L)	Membrane area (X3; cm^2)	Removal (%)			
				Measured		Predicted	
				Cd(II)	Pb(II)	Cd(II)	Pb(II)
**1**	90 (0)	50 (-1)	12.5 (-1)	75.8	93.0	79.7	92.4
**2**	90 (0)	150 (1)	12.5 (-1)	61.4	79.1	64.2	79.9
**3**	90 (0)	50 (-1)	37.5 (1)	95.3	99.2	92.5	98.4
**4**	90 (0)	150 (1)	37.5 (1)	90.9	96.1	87.1	96.7
**5**	60 (-1)	50 (-1)	25 (0)	88.6	92.1	88.5	93.1
**6**	60 (-1)	150 (1)	25 (0)	74.0	86.9	74.9	86.5
**7**	120 (1)	50 (-1)	25 (0)	93.0	98.9	92.1	99.3
**8**	120 (1)	150 (1)	25 (0)	84.6	92.6	84.7	91.6
**9**	60 (-1)	100 (0)	12.5 (-1)	75.1	82.9	71.4	82.5
**10**	60 (-1)	100 (0)	37.5 (1)	88.3	96.6	91.2	96.4
**11**	120 (1)	100 (0)	12.5 (-1)	83.0	90.4	80.1	90.6
**12**	120 (1)	100 (0)	37.5 (1)	92.1	99.3	95.9	99.7
**13**	90 (0)	100 (0)	25 (0)	91.4	97.2	91.4	97.2

Table S2: Regression statistics of Cd removal.

**Multiple R**	0.959743
**R Square**	0.921107
**Adjusted R Square**	0.684426
**Standard Error**	5.558102
**Observations**	13

Table S3: ANOVA of Cd removal process.

	**Df**	**SS**	**MS**	**F**	**Significance F**
**Regression**	9	1082.039	120.2266	3.891773	0.145428
**Residual**	3	92.6775	30.8925		
**Total**	12	1174.717			

TableS4: Regression statistics of Pb removal.

**Multiple R**	0.995425
**R Square**	0.99087
**Adjusted R Square**	0.963481
**Standard Error**	1.223043
**Observations**	13

Table S5: ANOVA of Pb removal process.

	**df**	**SS**	**MS**	**F**	**Significance F**
**Regression**	9	487.0321	54.11468	36.17695	0.006641
**Residual**	3	4.4875	1.495833		
**Total**	12	491.5196			

Table S6: Parameters and determination coefficients of the kinetic models for Cd(II) and Pb(II) adsorption on prepared nanofiber membrane.

**Initial concentrations (mgL^{-1})**	**50**		**100**		**200**	
	**Cd(II)**	**Pb(II)**	**Cd(II)**	**Pb(II)**	**Cd(II)**	**Pb(II)**
$q_{e,exp}$ (mg g^{-1})	245	251	462	486	866	907
Pseudo-1st-order						
$q_{e'cal}$ (mg g^{-1})	56	39.6	187	233	327	465
k_1(min^{-1})	0.035	0.026	0.039	0.024	0.034	0.029
R^2	0.93	0.96	0.96	0.97	0.96	0.95
Pseudo-2nd-order						
$q_{e,cal}$ (mg g^{-1})	258	249	472	478	897	915
k_2(min^{-1})	0.0281	0.0141	0.0102	0.0037	0.0055	0.0034
R^2	0.999	0.999	0.999	0.999	0.999	0.999

Table S7: Adsorption isotherm parameters for Cd (II) and Pb (II) adsorption on prepared nanofiber membrane.

**Temperature ºC**	**25 ºC**		**35 ºC**		**45ºC**	
	**Cd(II)**	**Pb(II)**	**Cd(II)**	**Pb(II)**	**Cd(II)**	**Pb(II)**
Langmuir isotherm						
q_m (mg g^{-1})	1030	1078	1098	1162	1149	1201
k_L(L mg^{-1})	0.11	0.29	0.16	0.36	0.31	1.39
R^2	0.999	0.999	0.998	0.999	0.998	0.999
Freundlich isotherm						
K_F(mg g^{-1})	168	264	195	311	269	466
$1/n_F$	0.48	0.42	0.47	0.44	0.39	0.33
R^2	0.998	0.998	0.998	0.999	0.999	0.999
